# Effective Connectivity Alterations of Directed Brain Network in Pediatric Patients With Spinal Cord Injury

**DOI:** 10.1002/cns.70720

**Published:** 2025-12-25

**Authors:** Yu Wang, Beining Yang, Ling Wang, Haotian Xin, Qunya Qi, Yulong Jia, Xianglin Guo, Weimin Zheng, Xin Chen, Fang Li, Qian Chen, Jubao Du, Jie Lu, Nan Chen

**Affiliations:** ^1^ Department of Radiology and Nuclear Medicine, Xuanwu Hospital Capital Medical University Beijing China; ^2^ Beijing Key Laboratory of Magnetic Resonance Imaging and Brain Informatics Beijing China; ^3^ Department of Radiology, Beijing Friendship Hospital Capital Medical University Beijing China; ^4^ Department of Radiology, Beijing Chaoyang Hospital Capital Medical University Beijing China; ^5^ Department of Rehabilitation Medicine, Xuanwu Hospital Capital Medical University Beijing China

**Keywords:** children, dynamic causal modeling (DCM), effective connectivity (EC), independent component analysis (ICA), resting‐state magnetic resonance imaging (rs‐fMRI), spinal cord injury (SCI)

## Abstract

**Aims:**

To investigate the alterations in effective brain connectivity in pediatric patients with spinal cord injury (SCI), to reveal the mechanism of brain network reorganization and to identify potential key targets for therapeutic neuromodulation interventions.

**Methods:**

This study enrolled 37 pediatric patients with SCI (24 with complete SCI, 13 with incomplete SCI) and 37 matched healthy controls. All participants underwent resting‐state functional MRI. Independent component analysis was conducted to identify intrinsic brain networks and obtain key regions of interest. Dynamic causal modeling (DCM) was applied to further analyze the effective connectivity (EC).

**Results:**

Patients with SCI showed significantly reduced connectivity between the default mode network (DMN) and the salience network (SAN). DCM revealed that the posterior cingulate cortex (PCC) was a key upstream regulator, exerting enhanced inhibitory influence on the medial prefrontal cortex, bilateral insula, and bilateral inferior parietal lobule. Subgroup analyses revealed that complete SCI was associated with increased excitatory drive from the DMN to the SAN, but enhanced inhibitory influence in the reverse pathway compared to incomplete SCI.

**Conclusion:**

The PCC is a pivotal node in post‐SCI brain reorganization, suggesting it as a potential neuromodulation target. The bidirectional DMN‐SAN regulatory imbalance is closely related to SCI severity.

## Graphical abstract

The PCC is a key upstream node in post‐SCI reorganization. Mechanistic differences between complete and incomplete SCI involve the bidirectional DMN‐SAN imbalance.

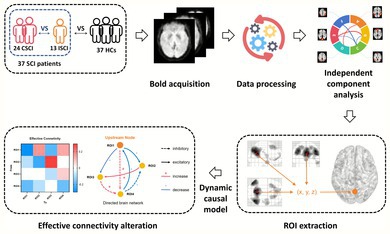

## 1 Introduction

Traumatic spinal cord injury (SCI) is a serious neurological disease that leads to sensorimotor dysfunction and profoundly affects patient quality of life. In recent years, an increasing number of studies have revealed that SCI can trigger extensive and complex reorganization of functional brain networks, which may play a key role in sensory motor function rehabilitation and secondary pathological injuries [1, 2, 3, 4]. Particularly, in pediatric patients with SCI, who are in a critical period of nervous system development and maturation, the pathophysiological mechanisms underlying the reorganization of the spinal and brain neural circuits following SCI may fundamentally differ from those observed in adults [5]. Furthermore, brain reorganization patterns in patients with complete (CSCI) and incomplete (ISCI) SCI do not necessarily follow the same mechanism [1]. These differences in reorganization may be closely related to the patients' rehabilitation potential. Previous studies have revealed multiple modes of mutual regulation among the functional networks of the human brain. A notable example is the triple‐network model, which posits that the salience network (SAN) exerts causal control over both the default mode network (DMN) and the central executive network (CEN), processes critically linked to both cognitive and executive control abilities [6, 7]. Altered interactions among these networks have been observed in various disorders, including Alzheimer's disease, depression, and obsessive‐compulsive disorder, and are associated with the occurrence of corresponding functional impairments [8, 9, 10]. However, how the interactions between multiple networks change and which regions dominate these changes in pediatric patients with SCI currently remains unclear. Clarifying the causal relationship between the interactions of each network may help to identify targets that play a major role in the reorganization of the entire network and guide the relevant treatment.

Current research into SCI has predominantly focused on analyzing resting state functional connectivity (FC), a measure of temporal correlations between spatially distant brain regions; however, this analysis cannot determine the direction of information flow or causal interactions between neural systems [11, 12]. Conversely, effective connectivity (EC) analysis quantifies the direct influence one neuronal system exerts on another, offering insight into the causal relationships within brain networks [13, 14]. Previous studies on EC in adults with SCI have partially revealed the mechanisms underlying motor imagery and psychotherapy rehabilitation [15, 16, 17], predominantly focusing on the effects of task states. Due to ongoing neurodevelopment in children, the impact of SCI on the interactions between brain networks may differ substantially from that observed in adults. Nevertheless, few studies have investigated the alterations in the directed network and EC in pediatric patients with SCI. Our study employed Dynamic Causal Modeling (DCM), a method grounded in a Bayesian framework, to infer the EC between brain regions and reveal their directional and causal properties [13, 18]. Unlike descriptive, correlation‐based methods (e.g., Granger Causality), DCM is a generative model that incorporates prior neurobiological knowledge to simulate the underlying neural dynamics. Technically, the DCM simulates the neuronal activity using a nonlinear dynamic differential equation model and then inversely estimates the EC parameters from the observed blood oxygen level‐dependent (BOLD) signal [19]. This approach thus provides a direct, nonlinear description of neural systems, resulting in a method that not only quantifies EC with high operational efficiency and accuracy [12, 18], but also serves as a unique tool for elucidating the mechanisms underlying neural network reorganization.

Currently, DCM is widely applied in the fields of stroke, mental diseases, and cognitive neuroscience [8, 20, 21], thereby providing unique perspectives for exploring the mechanisms underlying disease occurrence and treatment. However, their potential use in SCI research has not yet been fully explored. Thus, how brain regions affect each other after SCI, and whether the brain regions affect each other in the same way after CSCI and ISCI currently remain unclear. Since the brain functional reorganization after SCI is heterogeneous across studies, it is not appropriate to select regions of interest (ROIs) through other studies; therefore, we chose to adopt a data‐driven approach to initially obtain networks with significant FC alterations in SCI. As a data‐driven method, independent component analysis (ICA) can extract functional networks with physiological significance from functional magnetic resonance imaging (fMRI) data, such as the most classical DMN in resting‐state fMRI (rs‐fMRI), and has been consequently widely applied in various diseases [22, 23, 24].

In this study, we combined ICA with spectral DCM (spDCM): first, ICA was performed to extract classical brain network components that were significantly altered in patients with SCI, from which the main nodes were identified for subsequent analysis. Subsequently, spDCM was applied to investigate the EC between these nodes and to analyze the relationship between these alterations and clinical indicators. This approach is crucial for uncovering the mechanisms of cerebral neural remodeling following SCI and may reveal potential therapeutic targets for patients with SCI.

## 2 Materials and Methods

### 2.1 Participants

The study protocol was approved by the Medical Ethics Committee of Xuanwu Hospital, and written informed consent was obtained from the guardians of all participants. A cohort of 74 individuals was enrolled, comprising 37 pediatric patients with SCI and 37 typically developing (TD) children, who served as healthy controls (HCs). The inclusion criteria for patients were as follows: (1) patients with traumatic SCI, (2) age 6–12 years, (3) right‐handed, (4) injury duration exceeding 2 months, (5) no contraindications to MRI, and (6) no detectable brain abnormalities on conventional MRI. HCs were selected based on the following criteria: (1) age 6–12 years, (2) right‐handed, (3) age and sex matching with patients, (4) no contraindications to MRI, and (5) no detectable brain abnormalities on conventional MRI. The exclusion criteria for both groups were as follows: (1) any history of traumatic brain injury, psychiatric disorders, or substance abuse; (2) any structural brain abnormalities; and (3) poor image quality. Two rehabilitation specialists, Dr. Du and Dr. Li (with 29 and 11 years of experience, respectively) evaluated the motor and sensory functions of all pediatric patients using the International Standards for Neurological Classification of Spinal Cord Injury (ISNCSCI). Sensory scores were calculated by summing the segmental scores for the light touch and pinprick sensations. The neurological level of the lesion was determined using the American Spinal Injury Association (ASIA) Impairment Scale (available at: https://www.physio‐pedia.com/American_Spinal_Injury_Association_(ASIA)_Impairment_Scale). Table 1 presents the demographic characteristics of the 37 pediatric patients with SCI, including the etiology of injury, age at enrollment, duration of injury, sensory and motor scores, lesion level, and ASIA grade.

**TABLE 1:** Clinical data of 37 pediatric patients with spinal cord injury (SCI).

ID	Genders	Age at enrollment (years)	Duration of injury (months)	Etiology	Motor scores (0–100)	Sensory scores (0–224)	Level of lesion	ASIA grade
1	2	11	37	Backbend	50	128	T10–T12	A
2	2	12	77	Fall	50	128	T8–11	A
3	2	10	27	Backbend	50	144	T4–10	A
4	2	12	48	Backbend	50	128	T9–T10	A
5	2	11	45	Backbend	80	176	T6–L1	D
6	2	10	14	Backbend	50	144	T9–T12	A
7	2	6	36	Fall	80	188	T10–11	C
8	2	7	4	Backbend	50	136	T7–L1	A
9	2	7	25	Fall	50	152	T9–T11	A
10	2	10	5	Backbend	50	128	T8–T12	A
11	2	7	24	Backbend	50	128	T10	A
12	2	6	12	Fall	80	200	T4–T5	D
13	2	7	21	Fall	94	176	T8–11	D
14	2	8	25	Backbend	50	136	T10–L1	A
15	1	8	4	Strain	50	88	T3–7	A
16	2	8	12	Backbend	50	120	T9	A
17	2	10	36	Backbend	50	128	T4–9	A
18	2	9	5	Backbend	50	68	C7–T1	A
19	2	8	16	Backbend	50	128	T3/4–10	A
20	2	8	2	Backbend	74	176	T6	B
21	2	7	7	Backbend	60	144	T7–L1	C
22	1	9	35	Fall	50	128	T10–L1	A
23	2	9	42	Backbend	50	80	T2–3	A
24	1	7	46	Traffic accident	54	148	T3	C
25	2	11	36	Swim	50	132	T6	A
26	2	10	11	Backbend	70	144	T11	C
27	2	10	11	Backbend	90	172	T10–12	D
28	2	12	22	Backbend	50	136	T10–L1	B
29	2	9	8	Backbend	50	144	T4–L1	A
30	2	6	48	Traffic accident	50	156	T10	B
31	1	7	21	Backbend	50	120	T6	A
32	2	9	7	Backbend	84	184	T5–L1	D
33	2	8	24	Backbend	50	144	T10	A
34	2	12	24	Fall	34	80	C3–C7	A
35	2	9	75	Fall	50	128	T9	A
36	2	7	37	Kicking	50	132	T10–L1	A
37	1	6	9	Traffic accident	60	92	C7–T1	B

*Note:* The variable “gender” was set to 1 for males and 2 for females. Sensory scores are determined by summing the segmental assessments of light touch and pinprick sensations. The level of lesion refers to the neurological level. ASIA (American Spinal Injury Association) impairment scale: A: complete no sensory or motor function is preserved in sacral segments S4–S5; B: incomplete sensory but not motor function is preserved below the neurological level and extends through sacral segments S4–S5; C: incomplete motor function is preserved below the neurological level, and more than half of the key muscles below the neurological level have a muscle grade of < 3; D: incomplete motor function is preserved below the neurological level, and at least half of the key muscles below the neurological level have a muscle grade of > 3. Patients with SCI ASIA grade of A were defined as complete SCI, and patients with ASIA grade of B–D were defined as incomplete SCI.

### 2.2 Image Acquisition

The rs‐fMRI data were obtained using a Trio 3.0 T MRI scanner (Siemens, Erlangen, Germany) equipped with a 12‐channel head coil. All participants were given earplugs to minimize scanner noise. Participants were instructed to lie supine with their eyes closed, remain still, and avoid engaging in any specific thoughts during the procedure. Initial screening for brain abnormalities was performed using axial fluid‐attenuated inversion recovery sequences with the following acquisition parameters: repetition time (TR) = 8500 ms, echo time (TE) = 87 ms, matrix size = 256 × 256, inversion time (TI) = 2500 ms, flip angle = 150°, and slice thickness = 5 mm. The parameters for the echo‐planar imaging sequence were: 35 axial slices per volume (slice thickness = 3 mm, interslice gap = 1 mm), TR = 2000 ms, TE = 30 ms, flip angle = 90°, matrix size = 64 × 64, field of view (FOV) = 220 × 220 mm^2^, readout bandwidth = 2004 Hz/pixel, and voxel dimensions = 3.4 × 3.4 × 4.2 mm^3^. Each scan lasted 6 min, yielding 180 volumes per participant.

### 2.3 Data Preprocessing

All fMRI data were preprocessed using SPM12 (http://www.fil.ion.ucl.ac.uk/spm) and DPABI (Version 6.1, http://www.restfmri.net) within the MATLAB 2022b environment (MathWorks Inc., Natick, MA, USA). Initially, the first 10 time points of the fMRI data were removed, and a slice‐timing correction was performed with the middle slice as the reference. Next, all volumes were spatially realigned to correct for head motion using rigid‐body registration. Participants with head movements exceeding 2.5 mm in translation or 2.5° in rotation in any direction were excluded from further analysis. The realigned images were spatially normalized to a pediatric‐specific brain template for children aged 6–12 years in Montreal Neurological Institute (MNI) space (https://www.nitrc.org/projects/chn‐pd), with resampling to a 3 mm^3^ voxel size. The dataset was finally smoothed using a 6‐mm full width at half maximum (FWHM) isotropic Gaussian kernel to boost the signal‐to‐noise ratio.

### 2.4 Independent Component Analysis

Preprocessed fMRI data were decomposed into independent components (ICs) using the Group ICA of fMRI Toolbox (GIFT Version 4.0 b; http://icatb.sourceforge.net). Specifically, the ICA was conducted in three stages: (1) dimensionality reduction, (2) IC estimation, and (3) back‐reconstruction for each participant [25]. Initially, two‐stage principal component analysis was conducted for data reduction, resulting in 32 components based on all of the fMRI data. The number of components was determined using the minimum description length criterion [26]. The second stage involved estimating and identifying the most stable and reliable ICs. The Infomax algorithm [27] for Group ICA was applied in this step, which was repeated 100 times using the ICASSO algorithm [28, 29, 30]. Finally, the individual‐level component spatial maps were obtained through back‐reconstruction, with subject‐specific maps represented by Z‐scores, reflecting the intensity of connectivity within each IC [31]. The criteria for ICs identification included the following: (1) time courses dominated by low‐frequency fluctuations; (2) peak activation in gray matter regions; and (3) minimal spatial overlap with white matter, ventricles, vascular structures, or artifacts [31]. All the 32 ICs were identified with reference to the established network templates [32, 33]. All components were inspected independently by two experienced neuroradiologists (Dr. Lu and Dr. Chen, 28 and 15 years of experience, respectively), and discrepancies were resolved by a third (Dr. Chen N, 33 years of experience). This process yielded a final set of 19 valid components, all of which constituted subnetworks of larger functional networks. Internetwork connectivity analysis and between‐group comparisons were finally conducted, in which age and sex were regressed out as nuisance covariates, with Bonferroni correction applied for multiple comparison correction.

### 2.5 ROI Definition

Group‐mean maps of the DMN and SAN were generated by submitting the subject‐specific components to a one‐sample t‐test in SPM12, using a significance threshold of *p* < 0.001 at the voxel level (uncorrected) and *p* < 0.05 at the cluster level with family‐wise error (FWE) corrected. From these group‐level maps, peak coordinates were identified for 7 ROIs: the posterior cingulate cortex (PCC), medial prefrontal cortex (mPFC), left inferior parietal lobule (LIPL), right inferior parietal lobule (RIPL), left insula (LInsula), right insula (RInsula), and the anterior cingulate cortex (ACC). Each ROI was defined as an 8‐mm sphere centered on the peak activation within the main cluster of the respective network. Subject‐specific time‐series data were then extracted from these spherical ROIs for further analysis. Definitive MNI coordinates for each region are presented in Table 2.

**TABLE 2:** The detailed location information (MNI coordinates) for each ROI.

Name	Rough designation	MNI coordinates
DMN		
PCC	Posterior cingulate cortex	(−3, −63, 24)
mPFC	Medial prefrontal cortex	(−3, 39, −12)
LIPL	Left inferior parietal lobule	(−42, −69, 36)
RIPL	Right inferior parietal lobule	(45, −66, 33)
SAN		
LInsula	Left insula	(−42, 9, −9)
RInsula	Right insula	(42, 9, −15)
ACC	Anterior cingulate cortex	(−3, 30, −3)

Abbreviations: DMN, default mode network; MNI, montreal neurological institute; SAN, salience network.

### 2.6 EC Analysis Based on spDCM

EC analysis was performed using DCM12 implemented in SPM12. The analysis processes were as follows: (1) Establishment of individual‐level DCM and estimation of connectivity parameters [34]. Taking the MNI coordinates of the selected ROIs as the spherical centers and a radius of 8 mm, the time‐series signals were extracted to construct a DCM model with seven fully connected nodes (including self‐connections of each node, yielding a total of 49 connections). White matter and cerebrospinal fluid signals were extracted and regressed as covariates. Then Bayesian model reduction (BMR) was subsequently performed to determine the best model suitable for each subject [34]. (2) Estimation of the group‐level model and connectivity parameters was conducted using the parametric empirical Bayes (PEB) method [35]. The Bayesian model average of the connection parameters was obtained as the strength of the EC by calculating the weighted reliability of the connection parameters of the optimal model. In the DCM framework, the coupling parameters are described as ‘inhibitory’ or ‘excitatory’ based on their sign (negative or positive, respectively), reflecting the inhibitory or excitatory influence one neuronal population exerts on another at the modeled level of neural populations.

### 2.7 Statistical Analysis

The normality of all continuous data was assessed using the Shapiro–Wilk test. Based on the results, two‐sample *t*‐tests and the Mann–Whitney *U* test were used to examine group differences in normally and non‐normally distributed data, respectively. Two‐sample *t*‐tests were applied to analyze between‐group differences in age, while chi‐square tests were used for sex. The Mann–Whitney *U* test and Spearman's rank correlation were used for the duration of injury, motor scores, and sensory scores in patients with SCI. The Mann–Whitney *U* test was applied to compare the inter‐network connectivity between SCI patients and HCs after ICA (*p* < 0.05 was considered significant, and the resulting *p*‐values were corrected for multiple comparisons using Bonferroni correction). All of these analyses were performed using SPSS 25.0 (IBM Inc., Armonk, NY, USA). PEB analysis was performed using Bayesian posterior inference to test for differences in EC between patients with SCI and HCs. As PEB is a multivariate (Bayesian) general linear model (GLM) in which all model parameters were fit at once, no correction for multiple comparisons is required [36, 37]. The potential confounders of age and uneven sex distribution were regressed out in all models by including them as nuisance covariates. Statistical significance for EC findings was defined as a Bayesian posterior probability (Pp) > 0.95 [36].

## 3 Results

### 3.1 Demographic and Clinical Characteristics

In this study, a total of 74 individuals were enrolled, which included 37 pediatric patients with SCI (mean ± SD: 8.73 ± 1.87 years; 5 males, 32 females) and 37 HCs (mean ± SD: 8.84 ± 2.06 years; 9 males, 28 females). There were no significant differences in age and sex between the patient and HC groups, or between the complete and incomplete SCI groups. Within the SCI patients, no significant differences were found in terms of the duration of injury between the CSCI (mean ± SD: 28.25 ± 21.42 months, range: 4–79 months) and ISCI (mean ± SD: 17.54 ± 15.00 months, range: 2–46 months) groups (*p* = 0.104). However, the CSCI group exhibited significantly lower motor (*p* < 0.001*) and sensory scores (*p* = 0.001*) compared to the ISCI group. Table 3 summarizes the demographic information of patients with SCI and HCs.

**TABLE 3:** The demographic information of the patients with SCI and HCs.

	SCI (*n* = 37)	HCs (*n* = 37)	*p* (SCI vs. HCs)	CSCI (*n* = 24)	ISCI (*n* = 13)	*p* (CSCI vs. ISCI)
Age (years)	8.73 ± 1.87	8.84 ± 2.06	0.394	9.08 ± 1.67	8.08 ± 2.10	0.192
Gender			0.235			0.806
Male	5	9		3	2	
Female	32	28		21	11	
Duration of injury (months)	24.49 ± 19.88	—	—	28.25 ± 21.42	17.54 ± 15.00	0.104
Motor scores	57.32 ± 14.55	—	—	49.33 ± 3.27	72.08 ± 15.86	< 0.001*
Sensory scores	134.70 ± 31.36	—	—	123.67 ± 22.13	155.08 ± 36.30	0.001*

Abbreviations: CSCI, complete spinal cord injury; HCs, healthy controls; ISCI, incomplete spinal cord injury; SCI, spinal cord injury.

* Represents a significant difference.

### 3.2 Results of ICA

**FIGURE 1:**
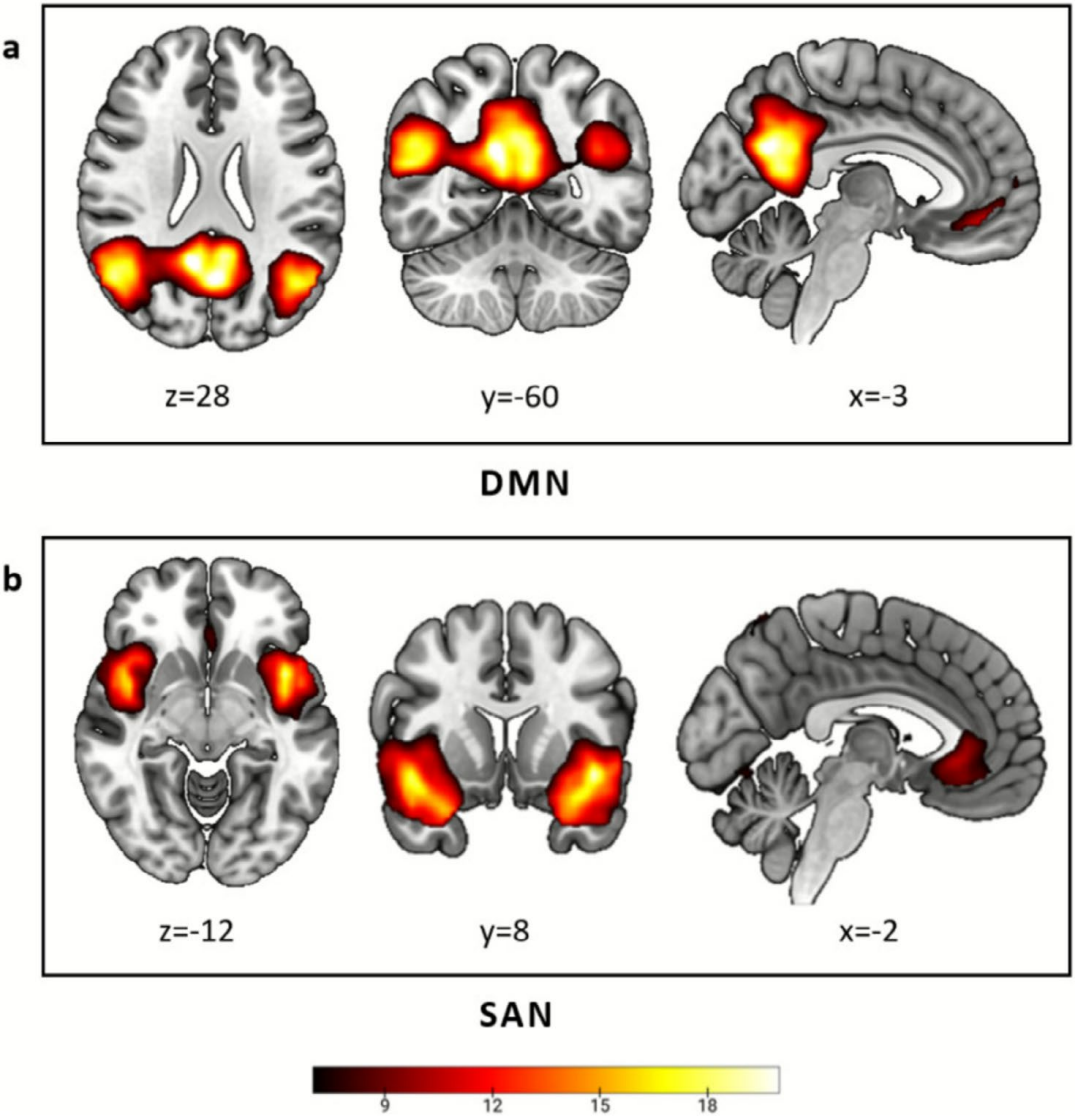
The distribution of the two components that showed significant differences between the spinal cord injury (SCI) group and the healthy control (HC) group in the results of the independent component analysis, namely the default mode network (DMN) (a) and the salience network (SAN) (b).

A total of 32 ICs were generated by the ICA. Nineteen ICs were selected for further analysis based on evaluation by three independent reviewers (Figure 1). Compared to HCs, the patient group showed a significantly decreased connection (*p* = 0.0003*, *T* = 3.766, Bonferroni correction) between IC9 and IC32, representing the DMN and the SAN, respectively.

### 3.3 Results of spDCM

#### 3.3.1 Group Mean EC

**FIGURE 2:**
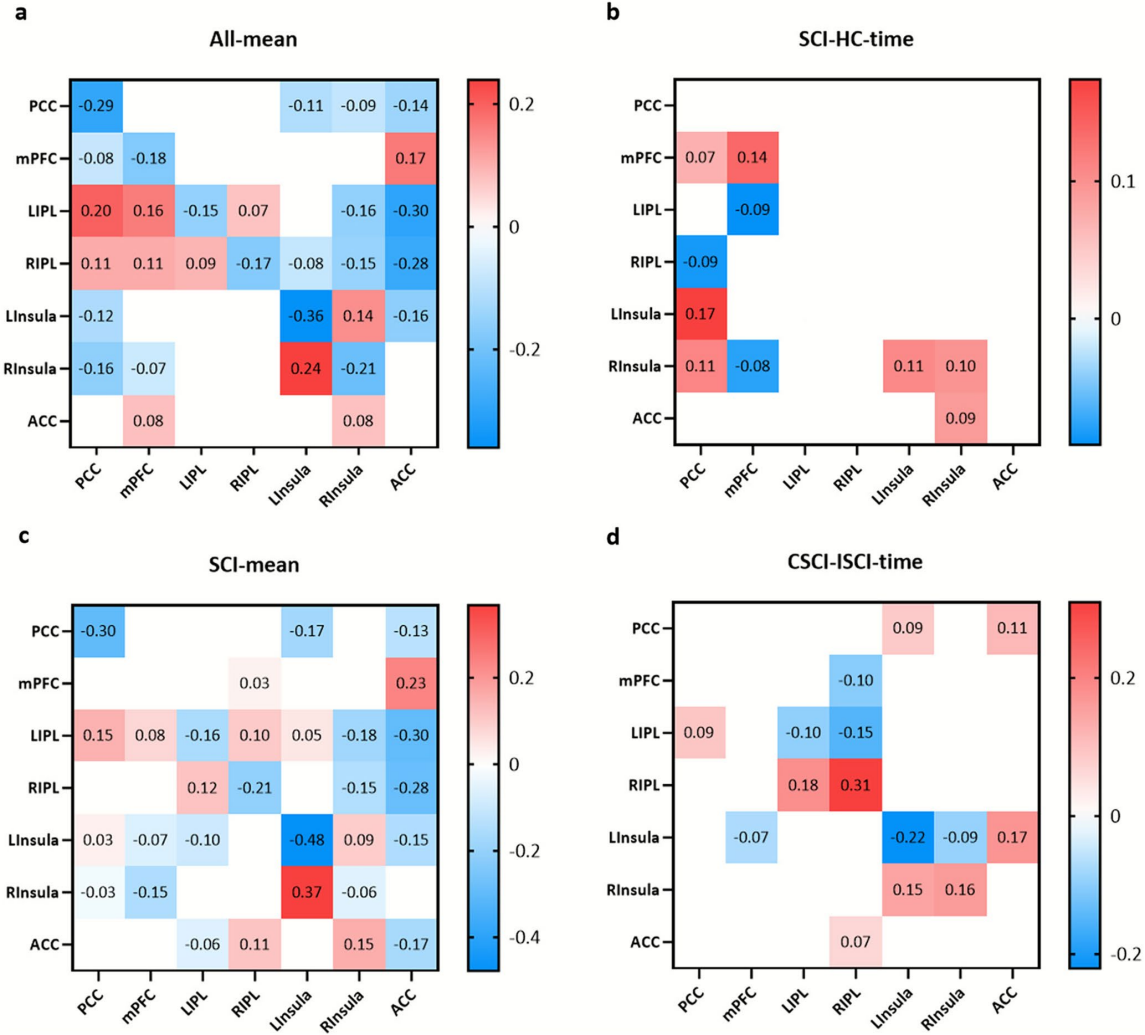
Heat map of group mean effect and time effect between groups. (a) shows the overall intrinsic connectivity of the SCI group and the HC group (group mean effect); (b) shows the change of effective connectivity (EC, time effect) between ROIs in SCI group compared with HC group; (c) shows the overall intrinsic connectivity of the CSCI and ISCI groups (group mean effect); (d) shows the change in EC (time effect) between each ROI between the CSCI and ISCI group. Colored areas represent EC with posterior probability (Pp) greater than 0.95. In (a and c), red represents excitatory intrinsic connectivity, and blue represents inhibitory intrinsic connectivity. In (b and d), red represents increased EC and blue represents decreased EC. SCI, spinal cord injury; HC, healthy control; CSCI, complete spinal cord injury; ISCI, incomplete spinal cord injury; PCC, posterior cingulate cortex; mPFC, medial prefrontal cortex; IPL, inferior parietal lobule; ACC, anterior cingulate cortex; L, left; R, right.

The mean effects of intrinsic connectivity in all participants and patients with SCI are presented in Figure 2. All seven regions of the ROIs exhibited the group‐mean effects of self‐inhibition in all participants. Furthermore, an inhibitory effect was observed between the DMN and SAN. Nearly identical group‐mean effects were found in the patients with SCI.

#### 3.3.2 Group Difference of EC

##### 3.3.2.1 Pediatric Patients With SCI Versus HCs

**FIGURE 3:**
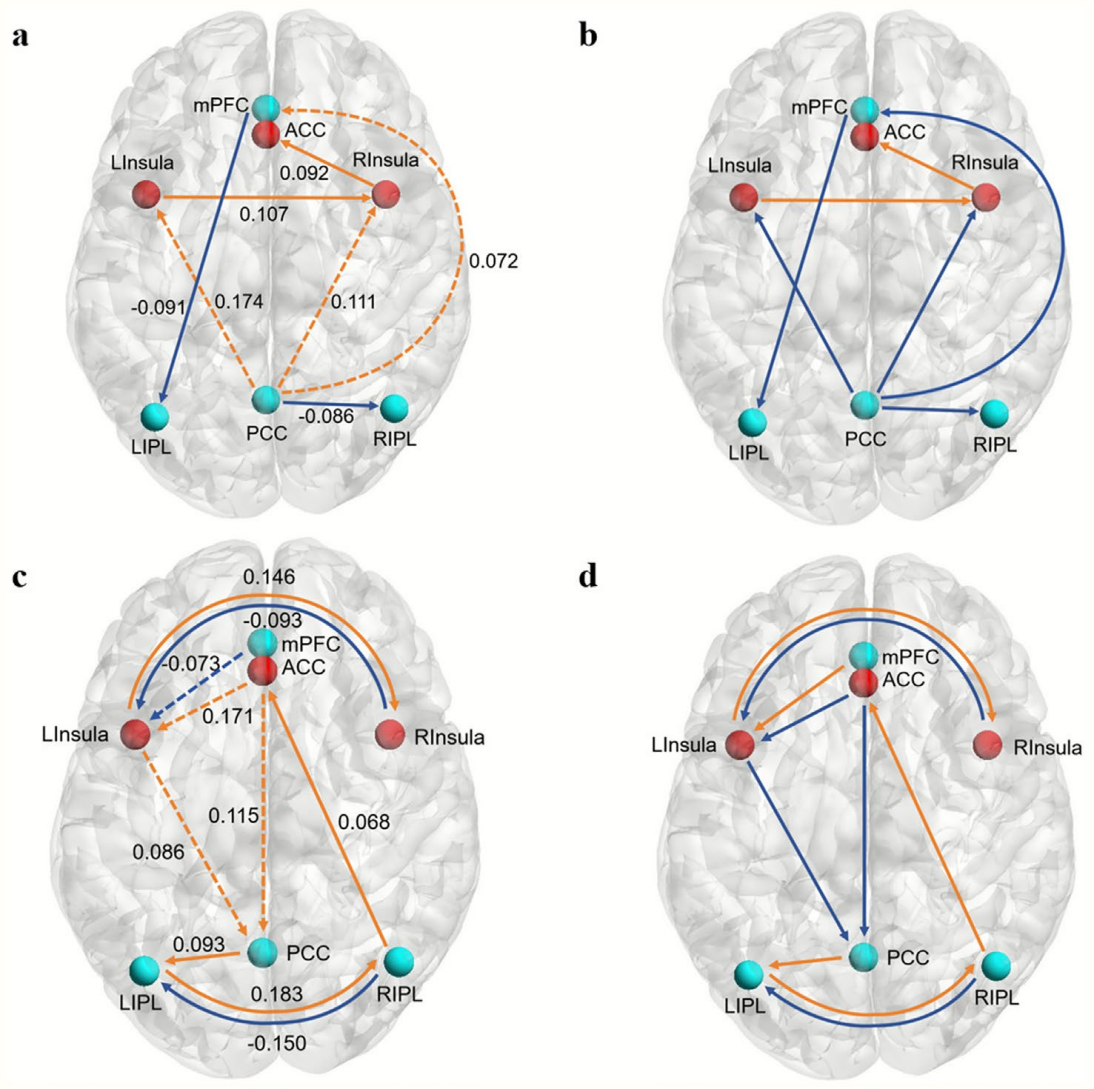
The brain diagram of the effective connectivity (EC) alterations of SCI patients. Nodes represent the brain regions, and edges represent the effective connectivity. (a and b) display changes in EC of spinal cord injury (SCI) patients compared to healthy controls (HCs), while (c and d) illustrate differences in EC between complete SCI (CSCI) and incomplete SCI (ISCI) patients. In (panels a and c), orange and blue arrows show increase and decrease in effect sizes (in units of Hertz (Hz)) while solid and dashed lines represent excitatory and inhibitory connections respectively; numerical values aside the lines indicate the magnitude of these changes. In (panels b and d), orange and blue arrows corresponds to increased and decreased EC, respectively.

We first investigated EC disruptions in all patients with SCI relative to the HCs. Overall, patients showed a greater inhibitory influence of the PCC on the mPFC and bilateral insula and reduced excitatory influence of the PCC on the right IPL and mPFC on the left IPL. In addition, patients showed an increased excitatory influence by the path way of LInsula–RInsula–ACC. The detailed findings of this comparison are shown in Figure 3a,b and Table 4.

**TABLE 4:** The effective connection changes of SCI patients compared to HCs and the effective connection differences between complete and incomplete SCI patients.

SCI‐HCs		CSCI‐ISCI	
Direction	Effect size (Hz)	Direction	Effect size (Hz)
PCC to mPFC	0.072	PCC to LIPL	0.093
PCC to RIPL	−0.086	LIPL to RIPL	0.183
PCC to RInsula	0.111	RIPL to LIPL	−0.15
PCC to LInsula	0.174	RIPL to ACC	0.068
mPFC to LIPL	−0.091	mPFC to LInsula	−0.073
LInsula to RInsula	0.107	LInsula to RInsula	0.146
RInsula to ACC	0.092	LInsula to PCC	0.086
		RInsula to LInsula	−0.093
		ACC to PCC	0.115
		ACC to LInsula	0.171

Abbreviations: ACC, anterior cingulate cortex; CSCI, complete spinal cord injury; HCs, healthy controls; IPL, inferior parietal lobule; ISCI, incomplete spinal cord injury; L, left; mPFC, medial prefrontal cortex; PCC, posterior cingulate cortex; R, right; SCI, spinal cord injury.

##### 3.3.2.2 Comparison of Patients With CSCI Versus ISCI Patients

Next, we examined whether patients with CSCI exhibited disruptions in EC similar to those observed in patients with ISCI. Compared to patients with ISCI, in CSCI, the ACC exhibited a heightened inhibitory effect on the PCC, either directly or via the ACC‐LInsula‐PCC pathway. Conversely, the PCC showed an increased excitatory influence on the ACC via the PCC‐LIPL‐RIPL‐ACC pathway. In addition, patients with CSCI exhibited a reduced inhibitory influence of the mPFC on the LInsula. The LInsula and LIPL exerted excitatory effects on the RInsula and RIPL, respectively, whereas the opposite was true for the inhibitory effect. Therefore, the mPFC has a facilitative effect on the RInsula via the mPFC‐LInsula‐RInsula pathway. For details, see Figure 3c,d and Table 4.

## 4 Discussion

In the present study, we first applied the ICA method and found that the static FC between the DMN and SAN was significantly reduced in pediatric patients with SCI. Although this discovery emphasizes the existence of large‐scale network disruptions, it remains descriptive, and therefore does not depict the direction of the impact of this changing relationship. To address this critical gap, we employed DCM, an EC approach that moves beyond correlations to infer causality. This advanced analysis not only confirmed the disruption, but also elucidated its specific mechanistic architecture; overall, we identified a widespread dysregulation of the EC, characterized by an enhanced inhibitory influence from the PCC (a DMN hub) onto key nodes including the mPFC, bilateral IPL, and insula (DMN and SAN hubs). This indicates that PCC is a potential upstream driver of network imbalance. Furthermore, DCM revealed distinct, severity‐dependent patterns of network reorganization between the CSCI and ISCI subgroups, characterized by a bidirectional regulatory imbalance between the DMN and SAN. These findings provide a more profound causal explanation for the observed reduction in static connectivity.

### 4.1 Inter‐Network FC Changes in Patients With SCI

ICA revealed significantly reduced FC between the DMN and the SAN in pediatric patients with SCI. This finding can be contextualized within the framework of the triple network model, which posits that interactions among the DMN, CEN, and SAN are crucial for understanding neurological disorders [9, 38]. The SAN normally facilitates dynamic switching between the self‐referential DMN and the executive‐oriented CEN [9, 39]. The chronic sensory deprivation and reduced environmental interaction in patients with SCI can diminish SAN engagement, thereby weakening its regulatory influence over the DMN and impairing the integration of internal cognition and external monitoring. This compromised network interaction may thereby contribute to executive dysfunction and potentially hinder motor rehabilitation, similar to patterns observed in other neurological conditions that influence cognitive‐motor integration [40].

### 4.2 EC Changes in Patients With SCI

This present study revealed abnormal EC patterns between the PCC and key regions in pediatric patients with SCI, including direct or indirect inhibitory effects on the mPFC, bilateral IPL, and insula. These findings highlight the critical role of the PCC in regulating information transfer between the DMN and other networks post‐SCI. As core DMN hubs, the PCC, mPFC, and IPL collectively support attention shifting, self‐referential processing, and cognitive control [41, 42]. The observed excessive intra‐network inhibition from the PCC may impair functions such as self‐reflection and memory integration, thus paralleling the mechanisms reported in depression, for which similar disruptions lead to rumination and altered self‐referential processing [42, 43].

Moreover, functional abnormalities in the PCC may disrupt information transmission across multiple brain networks, thereby influencing cognitive and emotional regulation [44]. In the present study, the observed excessive inhibition of the bilateral insula by the PCC in patients with SCI likely disturbed the dynamic balance between the DMN and the SAN, potentially contributing to emotional and cognitive deficits [45]. Similar dysconnectivity and PCC reorganization have been further reported in other neuropathological conditions, such as schizophrenia and Alzheimer's disease [46, 47, 48, 49]. Recent neuromodulation research has also indicated that transcranial alternating current stimulation can significantly alter the FC between the PCC and other regions, correlating with symptom improvement in patients with insomnia [50]. Similarly, deep brain stimulation targeting the PCC modulates hippocampal activity and improves behavior in patients with epilepsy [51]. These findings suggest that the PCC may serve as a key hub in large‐scale network reorganization following SCI, and as a potential target for precision treatment. However, further multicenter clinical validation is required.

In patients with SCI, interactions within the SAN are primarily characterized by excessive facilitation along the LInsula–RInsula–ACC loop. Comprising the bilateral insula and ACC, the SAN supports attentional switching, emotional regulation, and executive function [51]. Under typical conditions, the SAN helps to coordinate the dynamic balance between the DMN and the CEN in response to external stimuli [52, 53]. However, the enhanced intra‐network interactions may represent a compensatory mechanism following disrupted sensory input along the spinal‐thalamocortical pathway. Excessive compensation may result in over‐inhibition of the DMN or abnormal activation of the CEN, ultimately impairing attentional control and emotional regulation and reducing quality of daily life in patients [40].

### 4.3 Differences in EC Changes Between CSCI and ISCI Patients

Compared with ISCI patients, the DMN in CSCI patients exhibits abnormal excitatory influences on the SAN via two pathological pathways: PCC–LIPL–RIPL–ACC and mPFC–LInsula–RInsula. This was mainly related to the upstream nodes of the PCC and mPFC, which may imply disrupted attentional control due to DMN dysfunction. In healthy individuals, DMN–SAN interactions are tightly regulated to ensure appropriate attention allocation [40, 52, 53]. Conversely, in patients with CSCI, complete loss of sensory and motor inputs may induce compensatory DMN overactivity that pathologically influences the SAN, whereas partially preserved sensorimotor information may help to maintain normal SAN regulation of the DMN.

Patients with CSCI also showed enhanced inhibitory modulation from the SAN to the DMN through the ACC–LInsula–PCC pathway, possibly representing a compensatory negative feedback mechanism to counteract the aforementioned DMN‐induced interference with the SAN. The ACC, a key upstream node of the SAN along with the insula, typically plays a central role in allocating attentional resources, whereas the PCC serves as a core DMN hub involved in self‐referential processing [54]. Under conditions of complete sensorimotor loss, this strengthened inhibition from the SAN to the DMN may suppress task‐irrelevant internal thoughts, thereby preserving limited cognitive resources. Notably, dysregulated DMN‐SAN interactions have been reported in PTSD, and may reflect its pathophysiological mechanisms [55]. These findings indicate that rehabilitation protocols for patients with SCI should prioritize addressing abnormal DMN‐SAN interactions, with a particular emphasis placed on targeted neuromodulation of upstream hubs such as the PCC, mPFC, and ACC, which may offer therapeutic benefits across different injury severity grades.

### 4.4 Limitations

The relatively small sample size in this study may constrain its statistical power, particularly in subgroup analyses. However, we performed regressions with age and sex as nuisance covariates to mitigate these effects. The lack of behavioral or cognitive data (such as anxiety, depression, or cognitive scores) also prevents establishing associations between connectivity changes and functional deficits. Additionally, heterogeneity in injury levels and duration may introduce confounding effects. As a cross‐sectional design, this study also could not track temporal changes in network reorganization. Future longitudinal studies incorporating neuropsychological assessments and stratified injury analyses will be essential to validate these findings and clarify the behavioral significance of directed network changes in SCI.

## 5 Conclusion

This study employed a combined ICA and DCM approach to explore alterations in EC in children following SCI. Our findings demonstrate that the PCC is a key upstream node in the brain network changes in pediatric patients with SCI, potentially serving as a therapeutic intervention target and providing a theoretical basis for precise neural modulation. Finally, this study confirmed the existence of mechanistic differences between patients with CSCI and ISCI in terms of the bidirectional regulation imbalance between the DMN and SAN.

## Author Contributions

All authors contributed to the study conception and design. The material preparation, data collection, and analysis were performed by Yu Wang and Beining Yang. The first draft of the manuscript was written by Yu Wang and revised by Nan Chen. All the authors have read and approved the submitted manuscript.

## Funding

This study was supported by the National Natural Science Foundation of China (81871339 and 81271556), National Key Research and Development Program of China (2023YFF1204104), Beijing Municipal Natural Science Foundation (7113155), and Science Foundation of Beijing Municipal Commission of Education (KM201210025013).

## Ethics Statement

This study was approved by the Ethics Committee of Xuanwu Hospital, Capital Medical University (ethics no: [2020] 003), and was registered as a clinical trial (registration no: ChiCTR2000032793).

## Consent

The guardians of all participants enrolled in this study provided written informed consent for participation.

## Conflicts of Interest

The authors declare no conflicts of interest.

## Data Availability

The data that support the findings of this study are available from the corresponding author upon reasonable request.
